# Different patient subgroup, different ranking? Which quality indicators do patients find important when choosing a hospital for hip- or knee arthroplasty?

**DOI:** 10.1186/1472-6963-11-299

**Published:** 2011-11-03

**Authors:** Nicolien C Zwijnenberg, Olga C Damman, Peter Spreeuwenberg, Michelle Hendriks, Jany JDJM Rademakers

**Affiliations:** 1 NIVEL, Netherlands Institute for Health Services Research, P.O. Box 1568, 3500 BN Utrecht, the Netherlands; 2 Department of Public and Occupational Health and the EMGO Institute for Health and Care Research, VU University Medical Center, Amsterdam, the Netherlands

## Abstract

**Background:**

Patients are increasingly expected to become active, critical consumers in healthcare. They can use comparative healthcare information presented on websites to make informed choices for healthcare providers. However, the use of this information has been limited so far. An obstacle can be that the information is not perceived as relevant by patients. Presenting only the most important quality indicators might improve the usefulness of this information. The aim of this study was to explore which quality indicators different subgroups of patients find important when choosing a hospital for total hip arthroplasty (THA) or total knee arthroplasty (TKA).

**Methods:**

In this explorative, cross-sectional study, questionnaires were distributed to 265 patients who underwent or had to undergo THA/TKA. Participants were asked to rank the importance of three types of quality indicators: patient experience indicators, clinical performance indicators, and indicators about hospital services. We used random effects regression analyses to assess the relative importance of the indicators in different subgroups of patients.

**Results:**

110 patients (response rate 41.5%) who underwent or had to undergo THA/TKA participated. Conduct of doctors, the presence of procedures to prevent adverse effects of thrombosis and information about the specialist area of orthopaedists were the most important patient experience indicator, clinical performance indicator and indicator about hospital services, respectively. We found a few differences between patient subgroups in the importance attached to the quality indicators.

**Conclusions:**

This study provides a first insight into which quality indicators patients find important when choosing a hospital for THA/TKA, and shows that subgroups of patients differ in the value they attach to these indicators. More extended research is needed to establish the indicators that should at least be presented in succinct overviews of comparative healthcare information for patients choosing a hospital for THA/TKA.

## Background

In several Western countries, patients are expected to become active, critical consumers in healthcare, for example by searching information about their health and healthcare and by making deliberate healthcare choices. They are encouraged to actively choose between different health plans, hospitals, healthcare providers and treatment options [1-3]. To empower consumers and support their healthcare choices, comparative healthcare information is increasingly available on websites. The public disclosure of this information has emerged in several countries [3-5], including the Netherlands [6-8], and has resulted in a vast amount of information available to patients.

Although patients generally show interest in comparative healthcare information [9,10], several studies have demonstrated that the use of this information is limited [3,11-13] and that this information has only a small impact on patients' decision making [3,13,14]. A systematic review of Faber and colleagues (2009) [14] showed that patients are often unaware of the availability of comparative healthcare information, or they do not comprehend the information, or they think that the information is irrelevant and consequently ignore it in their decision process.

From decision making literature it is known that people can process and use only a limited amount of information [15,16]. For the presentation of comparative healthcare information this implies that presenting more information does not automatically support patients' choice, but will rather hamper an effective use of the information. Presenting only the most relevant information for patients is therefore essential [4,17]. Notwithstanding this need for only the most essential information, several researchers [18] have also challenged the 'one size fits all' approach of comparative healthcare information. Patients are incredibly diverse in terms of age, educational level, cultural background, health status and comfort with numerical displays [7,18]. Exploring which information is relevant for different patient subgroups when they make healthcare choices is therefore important.

### Different patients, different preferences?

Several studies have focused on the preferences of patients in healthcare [7,19-24]. Both technical and interpersonal quality have been shown to be important for patients [20]. In general, quality of the provided care, quality of staff, assortment of care, attitude and politeness of staff, and location of the healthcare organisation are important quality indicators for patients when making healthcare choices [19]. Patient preferences have been shown to be related to several socio-demographic and disease-related characteristics. For example, younger patients value having control over their healthcare and being involved in decision making, whereas older patients value a more 'traditional' physician who decides for them [21]. Another example is that breast cancer patients prioritize rapid access to care and diagnostics, while diabetics favour dignity and appropriate frequency of tests [25]. In addition, lower educated patients place great emphasis on continuity of care, whereas higher educated patients place great emphasis on the possibility of attaining a second opinion [21]. The challenge ahead is to present comparative healthcare information that suits different patients with different preferences. In this study, we assessed which comparative healthcare information is relevant for different patient subgroups choosing a hospital for total hip arthroplasty (THA) or total knee arthroplasty (TKA).

### Comparative information for Dutch THA/TKA patients

In the Netherlands, comparative healthcare information is currently available for different providers (such as health plans, hospitals, home care providers) and different treatments (such as breast cancer treatment, cataract surgery, and THA or TKA). One specific case to which the question of assessing the most relevant information for patients presently applies is information about THA/TKA. For this specific treatment, effort has been made for several years to assemble and disclose comparative healthcare information. Dutch THA/TKA patients are encouraged to use this information when choosing a hospital, especially because it concerns routine elective surgery for which people usually have sufficient time to search for information.

Different types of comparative healthcare information are available with regard to THA/TKA: patient experience indicators measured by the Consumer Quality Index (CQI: the Dutch standard for measuring patient experiences in healthcare) [26,27], indicators about hospital services and clinical performance indicators derived from hospital registrations. In 2009, an initiative was started to integrate these three types of information into comparative healthcare information disclosed at a consumer website (http://www.consumentendezorg.nl; Consumer and Healthcare). Keeping in mind that too much information can be overwhelming [17,28], a selection of these quality indicators was necessary as the amount of available information comprised in total 17 quality indicators (four patient experience indicators, six clinical performance indicators and seven indicators about hospital services).

### Research questions

The aim of this study was to examine which quality indicators patients find most important when choosing a hospital for THA/TKA, in order to get some first suggestions for essential indicators to be presented as comparative healthcare information. This study is the first step in studying the preferences of this specific patient group. For this purpose, the following research questions were addressed:

a. Which quality indicators do patients find most important when choosing a hospital for hip- or knee arthroplasty?

b. Are there differences between patient subgroups in terms of which quality indicators they find important?

## Methods

### Design

We used an explorative, cross-sectional design with a quantitative approach. THA/TKA patients had to rank various quality indicators according to importance when choosing a hospital. The ranking method is a simple and widely-used method to elicit patients' preferences [29-32]. Ethical approval of the study was not necessary as research by means of surveys that are not taxing and or hazardous for patients (i.e. the once-only completion of a questionnaire containing questions that do not constitute a serious encroachment on the person completing it) is not subject to the Dutch Medical Research Involving Human Subjects Act (WMO). Subjects were free to respond to the questionnaire and they were informed about the aim of the survey.

### Participants and data collection

The research population consisted of patients who underwent THA/TKA and patients who were on a waiting list for THA/TKA in several Dutch hospitals. Patients were invited for this study in different ways. First, patients were approached at orthopaedic departments of three general hospitals. These hospitals were selected via personal networks. Second, we posted calls on websites of patient organisations for orthopaedic patients, on websites of Dutch associations for senior citizens, and on the website of the Federation of Patients and Consumer Organisations in the Netherlands (NPCF). These patients could enrol in this study by contacting the research institute.

Data were collected through a survey in the period from May 2009 till August 2009. In total, we distributed 265 questionnaires. Each of the three general hospitals received 50 questionnaires. Doctors or nurses handed out the questionnaire when patients were discharged from the orthopaedic departments. The researchers sent 115 questionnaires by mail to participants who enrolled themselves in this study through the different websites. All participants had to return the questionnaire by mail. We aimed for at least 50 respondents (expected response rate: 20-50%).

### Procedures and Measures

The questionnaire contained three assignments in which participants had to rank the quality indicators from most important to least important when choosing a hospital for a total hip- or knee replacement. The first assignment included four patient experience indicators (CQI themes composed of questionnaire items through factor analysis) [26], the second assignment contained six clinical performance indicators and the final assignment contained seven indicators about hospital services. All quality indicators for which comparative healthcare information was available concerning THA/TKA were used in this study. We provided a short explanation for each quality indicator (see Additional file 1, Table S1).

The questionnaire further contained items about demographic characteristics: age, gender, perceived general physical health (poor - excellent; five-point scale) and level of education (no education - university; eight-point scale). We also asked whether participants had already undergone surgery or were on the waiting list for surgery. In addition, the survey contained six items that assessed patients' search and selection behaviour in healthcare (completely disagree - completely agree; four-point scale). These six items were developed and validated by Groenewoud (2008) [7] (see below).

Items of the search and selection behaviour scale

It doesn't matter too much to me where and by whom I am treated.

I don't want to invest too much time and energy in the choice process.

If I need care, I usually go the therapist/care facility to which my GP or specialist has referred me.

If I need care, I usually investigate thoroughly how, where and from whom I will receive the best treatment.

I have experience with the health care system and therefore know which therapist or care facility is best for me.

I think it's important to weigh possible treatments, therapists and care facilities against each other properly.

After recoding contra-indicative items, the scores on the six items were added up (range 6-24) and a higher score on this scale represented a more extensive search and selection behaviour in the care process. The scale showed high reliability (α = .77) and the mean score was 18.5 (95% CI 17.9-19.2). Looking at the possible scale range, this indicates a relatively active search and selection behaviour of the participants. Finally, the questionnaire contained an open-ended item in which participants could report any indicators missing in this survey that they would use when choosing a hospital.

### Data analyses

Data were analysed using STATA version 10.1 and MLwiN version 2.02. First, for each participant, scores were appointed to every indicator in each assignment. The most important indicator received a score that was equal to the amount of indicators in the assignment; the least important indicator received one point. For example, when a participant had to rank four indicators (e.g. the patient experience indicators), the indicator ranked most important received four points, the second indicator received three points, the third indicator received two points and the indicator ranked least important received one point. When a participant had not ranked every indicator in an assignment, only indicators that were ranked received points equally to the position on which it was ranked. Subsequently, an overall mean was calculated for every indicator in each assignment. The higher the mean, the more important this indicator was from the perspective of the participants.

We compared different patient subgroups as defined by demographic variables, by search and selection behaviour and by stage of healthcare (see Table 1). Subgroups were generated by dichotomizing all these variables and where possible, subgroups equal in size were used in the analyses. For example, the median score was used to compose patient subgroups differing in age and in search and selection behaviour.

**Table 1:** Participants' characteristics and composition of patient subgroups

Variable	This study N = 110		Measurement CQI Hip Knee (2009) N = 5.163
	**N**	**%**	**%**
Age			
up to 65 years	57	52	32
65 years and older	52	48	68
Gender			
male	36	33	33
female	73	67	67
Perceived health status			
poor to reasonable health	22	20	22
good to excellent health	87	80	78
Level of education^			
lower educational level	49	45	86
higher educational level	60	55	14
Healthcare stage			
on waiting list for surgery	10	11	-
underwent surgery/again on waiting list for new surgery	84	89	-
Search and Selection behaviour			
less extensive (score ≤ 19)	60	55	-
more extensive (score > 19)	49	45	-

^ Level of education: Low: primary school, lower level of secondary school, lower vocational training or intermediate vocational training. High: higher level of secondary school, higher vocational training or university.

To assess the differences in rankings between patient subgroups, we analysed the mean scores and the between-participants variances for every patient subgroup, using random effects regression analysis [33]. This type of linear regression allowed for the simultaneous estimation of the mean score and a between-participants variance for every indicator in the assignment. Traditional regression analysis would be inadequate, because it would assume that for every indicator the variance was equal. The difference in mean score between patient subgroups for every indicator in the assignments was tested by adding an interaction term based on the indicator-variable (e.g. patient experience indicator) and the subgroup-variable (e.g. gender). The regression coefficient showed the difference in ranking between the two patient subgroups (e.g. men vs. women). For the between-participants variance, for every subgroup a separate variance was estimated and these were compared using a contrast (Wald) test [33,34]. Results were deemed statistically significant at the 1% level (p < 0.01).

## Results

### Characteristics of the participants

One hundred and ten THA/TKA patients (response rate of 41.5%) participated (see Table 1). Of the participants, 83.6% enrolled themselves by responding to one of the calls on websites. The average age of the participants was 64 years (SD = 10.3) and the majority was female (67%). Most participants rated their own health positively; good (58%) or very good (22%). A large group of participants graduated from higher level of secondary school or a higher level of education (55%). Only ten percent of the participants had not yet undergone surgery.

To get some idea of the representativeness of our study population, we compared the characteristics of our participants to the characteristics of a larger population of THA/TKA patients. These data were derived from the 2009 nation-wide measurement with the Consumer Quality Index Hip Knee Questionnaire (CQI Hip Knee) among 8, 675 patients who underwent THA/TKA. The response rate in this measurement was 67% (N = 5, 163) [35]. Gender and perceived health status of both groups were comparable. However, participants in the current study were younger (M = 64 years vs. M = 68 years) and had a higher educational level than participants of the measurement with the CQI Hip Knee-survey.

### What do THA/TKA patients find most important?

In Table 2 the mean importance scores and variances of the different quality indicators are presented. As regards the *patient experience indicators *(CQI themes), participants ranked the conduct of doctors as most important (M = 3.76). The most important *clinical performance indicator *for participants was the presence of procedures to prevent adverse effects of thrombosis (M = 4.38). Whether orthopaedists have one or more specialist area was ranked as most important *indicator about hospital services *(M = 5.46). Participants were more homogeneous in their rankings of patient experience indicators compared to the rankings of indicators about hospital services and clinical performance indicators (see variances Table 2).

**Table 2:** Mean score and variance of the quality indicators

Assignment	Quality indicator	Mean	VAR
1. Patient experience indicators (CQI themes)			
	Conduct of doctors	3.76	0.36
	Pain control	2.46	0.74
	Conduct of nurses	2.42	0.51
	Information about new medication	1.34	0.45

2. Clinical performance indicators			
	Procedures to prevent adverse effects of thrombosis	4.38	1.69
	Information provision before surgery	4.35	2.71
	The occurrence and prevention of deep wound infections	4.13	2.07
	Registration of complications related to THA/TKA	3.16	2.14
	Transfusion of homologous blood	2.98	2.29
	National registration of orthopaedic implants	1.97	1.97

3. Indicators about hospital services			
	Specialist areas of orthopaedist	5.46	3.12
	Information provision approach	4.77	2.68
	Contact with hospital after surgery and hospital discharge	4.52	2.12
	Number of performed total knee- or hip replacements among adults in a year	4.40	4.06
	Number of orthopaedists in the hospital	3.84	3.03
	Group-hospital admission	3.19	3.16
	Number of performed total knee- or hip replacements among children in a year	1.80	1.30

Mean CQI themes: range 1-4; Mean clinical performance indicators: range 1-6; Mean indicators about hospital services: range 1-7.

### Differences in mean scores and ranking between patient subgroups

Tables S2, S3 and S4 (see Additional file 1, Table S2, S3 and S4) show the mean scores on the different quality indicators for the different patient subgroups. On two quality indicators patient subgroups differed. Men ranked information about new medication (patient experience indicator) as more important than women (see Additional file 1, Table S2). Participants with a higher educational level ranked the number of yearly performed total knee- or hip replacements among adults (indicator about hospital services) as more important than participants with a lower educational level (see Additional file 1, Table S4).

When we look at the ranking of the clinical performance indicators, we saw that younger patients, men and patients with a lower educational level ranked information provision before surgery as most important instead of the presence of procedures to prevent adverse effects of thrombosis, which was ranked overall as most important (see Additional file 1, Table S3). However, no significant differences on mean scores for these two indicators and the other clinical performance indicators were found between patient subgroups. Concerning the ranking of the other indicators, every patient subgroup ranked the conduct of doctors as the most important patient experience indicator (see Additional file 1, Table S2) and the specialist area of orthopaedists as the most important indicator about hospital services (see Additional file 1, Table S4).

### Variance scores: homogeneous or heterogeneous subgroups?

Tables S2, S3 and S4 (see Additional file 1, Table S2, S3 and S4) also present the between-participants variances on each quality indicator for the different patient subgroups. A few significant differences in variance scores were found. Conduct of doctors (patient experience indicator; see Additional file 1, Table S2) and the number of yearly performed total knee- or hip replacements among children (indicator about hospital services; see Additional file 1, Table S4) were indictors on which subgroups mainly differed in variances (i.e. differences in at least two patient subgroups). Additionally, significant differences in variance scores were mainly found (i.e. differences on two quality indicators) between patients with a different healthcare stage and between patients with a different perceived health status.

### Other important choice aspects

Sixty-five participants (59%) filled out the open-ended question in which they could report indicators that they thought were missing in this survey, but that they would use when choosing a hospital. Some indicators were mentioned several times by participants: distance to the hospital/accessibility, waiting times, reputation of the hospital and facilities of the hospital (e.g. rehabilitation and physical therapy options, recreational space, extended visiting hours). Some participants also mentioned the importance to take the experiences of others (e.g. other patients/general practitioner) into account.

## Discussion

This study explored which quality indicators patients find most important when choosing a hospital for THA/TKA and whether patient subgroups differed in which indicators they find important. By means of ranking indicators, patients indicated the importance of different patient experience indicators (CQI themes derived from the Dutch Consumer Quality Index questionnaire), clinical performance indicators, and indicators about hospital services. We demonstrated that patients perceived the conduct of doctors, the existence of procedures to prevent adverse effects of thrombosis and information about the specialist area of orthopaedists as the most important patient experience indicator, clinical performance indicator and indicator about hospital services, respectively. When presenting comparative healthcare information, it seems that these quality indicators are essential to incorporate. Our results also showed that patients of different subgroups and patients within subgroups sometimes differed in the importance attached to certain quality indicators. Although implementation can be difficult, website builders could consider supplementary options to present tailored comparative healthcare information. Hopefully, this will stimulate the use of comparative healthcare information by patients, which is limited up to now [3].

Due to the explorative nature of this study, definite conclusions as to which indicators to present cannot be drawn. More extended research based on the current results is needed to select the indicators to be presented for THA/TKA patients. Subsequent studies should preferably use experimental methods and include a larger group of patients to further investigate the perceived importance of the various quality indicators.

### Discussion of the results

Although in this study no direct comparison was made between the three different types of quality indicators, it seems that both interpersonal (i.e. conduct of doctors) and more technical aspects (i.e. prevention of adverse effects of thrombosis, specialist area of orthopaedists) are important for patients, which is in line with research of Wensing et al. (1998) [20]. Previous research has found mixed results concerning healthcare preferences: in some studies it was found that patients value the technical aspects more than interpersonal skills and service aspects [7,23], whereas other studies showed the opposite pattern [25,36]. The type of research, the patient group involved and the kind of healthcare choice may all affect patients' priorities. Evidence that preferences change over time [37] and the theory of constructed preferences [38], addressing that patient's preferences are often constructed during the decision making process, are reasonable explanations for the differences in findings between studies. That patients' priorities differ from person to person and can change from time to time, shows the complicated process to provide relevant comparative healthcare information that suits different patients.

The fact that patients can value different types of information as important (e.g. interpersonal and technical aspects), can increase the cognitive burden to make a deliberate hospital choice [16,28]. Patients have to weigh different indicators and have to make trade-offs in their choice. The decision making process will always require difficult cognitive processes, especially when contradictory information is involved. It is known that tailoring information can ease the cognitive burden as less information processing is required, because only the relevant aspects have to be taken into account in the decision making process [16].

Distance to the hospital, reputation of the hospital, waiting times, hospital facilities (e.g. revalidation options, extended visiting hours) and experiences of others were mentioned as other important indicators when choosing a hospital, which is in line with previous studies [22,39-42]. The integration of this kind of information into comparative healthcare information could thus be considered. However, the amount of information on web pages needs to remain manageable for patients. We would therefore advocate further research about the value of these indicators for patients' choice.

Our results show that patients' characteristics may to some extent determine which quality indicators patients find important, which corresponds to findings from previous research [21,42-44]. Gender and educational level of THA/TKA patients influenced the importance attached to the indicators information about new medication and the number of yearly performed total knee- or hip replacements among adults. In addition, age, gender, and educational level affected the value attached to information provision before surgery and the presence of procedures to prevent adverse effects of thrombosis. Although no significant differences were found for this latter result, - perhaps as a result of our small sample size -, these results indicate that different patients can have different preferences, which would challenge the 'one size fits all' approach of comparative healthcare information.

An interesting finding is that higher educated patients ranked the yearly performed total hip- or knee replacements among adults as more important than lower educated patients. The number of performed surgeries often is associated with the outcome of the surgery, by influencing the complication or mortality rates [45-47]. After all, the more experience doctors have, the less chance medical mistakes occur. The question rises whether people with a lower educational level interpret this indicator as such. More qualitative research is needed to provide insight into how people interpret this kind of quality indicators.

Given these results, an interesting thought is to what extent the perceived relevance of indicators by (future) patients should drive the selection of quality indicators for comparative health care information. One could argue that crucial information, like the number of performed surgeries, should be available for every patient even if patients do not perceive it as one of the most important indicators. When such crucial information is only provided to patients who attach value to it, this may possibly result in inequities in the healthcare system. Ideally, comparative healthcare information consists of a mixture between indicators perceived as important by patients, and crucial indicators known to be related to treatment outcomes perceived as important by experts.

A few significant differences in between-participants variance scores were found between subgroups, indicating that individuals *within *some patient subgroups also were less unanimous about the importance of certain quality indicators. It was notable that patients with a good to excellent health and a low educational level were less unanimous about the importance of conduct of doctors, which overall was ranked as the most important patient experience indicator. That individuals in some patient subgroups differed about the importance of indicators, stresses the relevance of a tailor-made presentation approach for comparative healthcare information [16,48], but also shows the difficulty to create this kind of information.

### Strengths & Limitations

An important feature of this study is that participants *ranked *different quality indicators instead of *rated *the importance of these indicators. By ranking indicators, patients must explicitly weigh what they find more and less important. By rating indicators, in contrast, patients can rate every indicator as equally important. So, by using the ranking method, the results provide more insight into what patients find most important. Another strength of this study is that we asked participants which quality indicators they find important *when choosing a hospital*, rather than we focused on which quality indicators they find important in general. As a result, the findings are more likely to improve comparative healthcare information in terms of relevance for patients' hospital choice. A final strength is that we included real patients who face or have faced a real hospital choice.

Although this explorative study adds to our understanding of what patients find important when choosing a hospital for THA/TKA, it also has several important limitations. First, the small sample size decreased the statistical power of our study and, combined with the multiple tests performed, this may lead to potential sources of both systematic and random errors. By using a P of 0.01, however, the chance that results are capitalizing on chance only is smaller.

Second, there are some limitations related to the representativeness of the sample. The majority of the participants had enrolled themselves in this study. Given this self-selection, participants of this study may be more interested in the topic of comparative healthcare information than the average THA/TKA population. When background characteristics of our participants were compared to participants of a nation-wide study using the CQI Hip Knee Questionnaire, it appeared that our participants were younger and had a higher educational level than the participants of the CQI Hip Knee-survey. The majority of participants also consisted of patients who had already undergone surgery. Patients who still have to undergo surgery may evaluate other quality indicators as important, because the phase or severity of the disease can determine patients' preferences [7]. In sum, it is unclear to what extent the results can be generalized to a larger group of THA/TKA patients.

Finally, besides the usual limitations inherent to an explorative study (e.g. potential for spurious relationships), there are some other methodological issues. The use of a ranking method forces people to make choices and this creates a hypothetical situation, since people can decide in real life to make no choice at all. In addition, by using separate ranking assignments for the three types of quality indicators, it was not possible to compare across the three dimensions of the quality indicators. The use of different ranking assignments, however, was a deliberate choice to decrease the cognitive burden for participants to fulfil the assignments. At last, this study was limited to three types of quality indicators. Other indicators also can be important for patients when choosing a hospital, as was illustrated by the answers of participants to our open ended question. Taking all these limitations into account, caution is warranted by the interpretation and generalization of the results.

### Implications

Despite the explorative nature of our study, our findings are already informative and of importance for builders of websites who develop comparative healthcare information for patients in general and, more specifically, for THA/TKA patients. Providers of comparative healthcare information often select the indicators that will be presented themselves, without taking the needs and wishes of patients systematically into account. However, involving patients, the users of the information, in the process of designing information is essential in making comparative healthcare information more relevant.

To some extent, our recommendations seem contradictory: on the one hand we advocate to only present concise information, on the other hand we suggest the presentation of tailored information. However, both aims can simultaneously be accomplished. This study gives a first impression of which quality indicators are most important from the patient's perspective: the conduct of doctors, the existence of procedures to prevent adverse effects of thrombosis, and the specialist area of orthopaedists. Making use of a succinct overview on the first web page for this kind of information will prevent that THA/TKA patients feel overwhelmed by the information provided. When considering supplementary tailored information about THA/TKA, the use of deep-linking and selection tools can be considered [5,28,49]. An option to consider is that patients themselves select the quality indicators they find important when choosing a hospital. The number of selected indicators can differ from person to person, adjusted to someone's own wishes. In this way, patients have an active role in their own information supply. The challenge ahead is to create a balance between the provision of concise, crucial information on the one hand, and to create supplementary tailored information that suits the target group on the other hand.

For future research we would recommend to examine which quality indicators are important for other patient groups when choosing a healthcare provider, in order to develop relevant comparative healthcare information for them as well. In our opinion, using different methods to elicit patient preferences is preferable. The use of the ranking method to explore patients' preferences is a good starting point, complemented with other research (e.g. discrete choice experiment or qualitative research) to profoundly explore patients' preferences. Although the central focus of this study concerned which aspects have to be presented for consumers, we acknowledge that the presentation approach is just as important [8,16] for making comparative healthcare information successful.

## Conclusions

This study gives a first impression of which quality indicators could be presented as comparative healthcare information for patients choosing a hospital for THA/TKA. However, extended research to validate these results is required.

Patients differed in the importance attached to a few quality indicators when choosing a hospital for THA/TKA. Although the implementation can be difficult, presentation approaches that tailor information could be considered. In this way, comparative healthcare information can be made more relevant and may motivate patients to use this information in their hospital choices.

## Competing interests

The authors declare that they have no competing interests.

## Authors' contributions

NZ performed the data collection and data analysis. She interpreted the data and drafted the manuscript. OD designed the study and contributed to the data collection and draft versions of the manuscript. PS performed the data analysis. MH contributed to the conception and design of the study and helped to draft the manuscript. JR contributed to the conception and design of the study as well as the draft versions of the manuscript. All authors read and approved the final manuscript.

## Pre-publication history

The pre-publication history for this paper can be accessed here:

http://www.biomedcentral.com/1472-6963/11/299/prepub

## Supplementary Material

Additional file 1 **Table S1**- Explanation of quality indicators. Table in which a description of every quality indicator is provided. **Table S2 **- Mean scores and variances of the patient experience indicators for the different patient subgroups. Table in which the results of the analysis for the patient experience indicators are provided. **Table S3 **- Mean scores and variances of the clinical performance indicators for the different patient subgroups. Table in which the results of the analysis for the clinical performance indicators are provided. **Table S4 **- Mean scores and variances of the indicators about hospital services for the different patient subgroups. Table in which the results of the analysis for the indicators about hospital services are provided. Click here for file
