# Supplementary material for: Different patient subgroup, different ranking? Which quality indicators do patients find important when choosing a hospital for hip- or knee arthroplasty?
Source: BMC Health Serv Res. 2011 Nov 3;11:299. doi: 10.1186/1472-6963-11-299 (PMC3268112; doi:10.1186/1472-6963-11-299)
Supplement: Additional file 1 — Table S1- Explanation of quality indicators. Table in which a description of every quality indicator is provided. Table S2 - Mean scores and variances of the patient experience indicators for the different patient subgroups. Table in which the results of the analysis for the patient experience indicators are provided. Table S3 - Mean scores and variances of the clinical performance indicators for the different patient subgroups. Table in which the results of the analysis for the clinical performance indicators are provided. Table S4 - Mean scores and variances of the indicators about hospital services for the different patient subgroups. Table in which the results of the analysis for the indicators about hospital services are provided. [file 1472-6963-11-299-S1.DOC]

Table S1- Explanation of quality indicators

| **Quality indicator** | | **Explanation for respondents** |
| --- | --- | --- |
|  | Conduct of doctors | Indicates how doctors and nurse practitioners have contact with patients, for example their politeness, careful listening, and clear explanations. |
|  | Pain control | Indicates how pain control in the hospital is arranged. For example, to what extent pain is under control, and whether everything possible is done to help the patient with his/her pain. |
|  | Conduct of nurses | Indicates how nurses have contact with patients, for example their politeness, careful listening, and clear explanations. |
|  | Information about new medication | Indicates how information about new medication is dealt with. For example, whether professionals tell about the use of new medication, and whether potential side effects are clearly explained. |
|  |  |  |
|  | Procedures to prevent adverse effects of thrombosis | Indicates how a hospital tries to prevent these adverse effects of thrombosis. For example, whether a hospital uses guidelines or blood thinners. Thrombosis is common after a total hip- or knee surgery. |
|  | Information provision before surgery | Indicates whether patients receive written or visual information before surgery. |
|  | The occurrence and prevention of deep wound infections | Indicates how often deep wound infections occur in hospitals and how hospitals deal with antibiotics. The provision of antibiotics prevents deep wound infections. |
|  | Registration of complications related to THA/TKA | Indicates whether a hospital registers occurring complications after surgery. |
|  | Transfusion of homologous blood | Indicates how a hospital deals with transfusion of homologous blood. Homologous blood is blood from another person and transfusion is risky because of the chance of infections and the chance of human mistakes (switch of blood). Transfusion of homologous blood is sometimes necessary, when the loss of blood by a patient during surgery is high. |
|  | National registration of orthopaedic implants | Indicates whether the hospital participates in the national registration of orthopaedic implants. By doing this, the quality of care related to implants is monitored and can be improved. |
|  |  |  |
|  | Specialist areas of orthopaedist | Indicates whether there are orthopaedists employed in the hospital with one or more specialist areas, such as hip dysplasia teenagers, children's orthopaedics, revision surgery, complex spine surgery or tumour surgery |
|  | Information provision approach | Indicates in which way patients receive information about orthopaedic surgery. For example, briefings, written information material, information via the website of the hospital and information via patient associations |
|  | Contact with hospital after surgery and hospital discharge | Indicates whether patients can have contact with the hospital by email or phone after surgery and hospital discharge |
|  | Number of performed total knee- or hip replacements among adults in a year | Indicates how many adults (18 and older) underwent surgery for a total hip replacement or total knee replacement |
|  | Number of orthopaedists in the hospital | Indicates how many orthopaedists are employed in the hospital |
|  | Group-hospital admission | Indicates whether patients are hospitalised in groups and these patients underwent surgery on the same day. The rehabilitation process also occurs in groups. For example, Joint Care or Joint Progress. |
|  | Number of performed total knee- or hip replacements among children in a year | Indicates how many children (under 18) underwent surgery for a total hip replacement of total knee replacement. |

# Table S2 - Mean scores and variances of the patient experience indicators for the different patient subgroups

| **Patient subgroup** | **Conduct of doctors** | | **Pain control** | | **Conduct of nurses** | | **Information about new medication** | |
| --- | --- | --- | --- | --- | --- | --- | --- | --- |
|  | M | VAR | M | VAR | M | VAR | M | VAR |
| Search and Selection behaviour |  |  |  |  |  |  |  |  |
| less extensive | 3.80 | 0.31 | 2.39 | 0.72 | 2.43 | 0.54 | 1.36 | 0.46 |
| more extensive | 3.71 | 0.43 | 2.57 | 0.76 | 2.40 | 0.47 | 1.31 | 0.44 |
| Age |  |  |  |  |  |  |  |  |
| up to 65 years | 3.78 | 0.38 | 2.45 | 0.66 | 2.47 | 0.53 | 1.27 | 0.32 |
| 65 years or older | 3.75 | 0.34 | 2.48 | 0.85 | 2.35 | 0.48 | 1.43 | 0.59 |
| Gender |  |  |  |  |  |  |  |  |
| male | 3.74 | 0.41 | 2.37 | 0.83 | 2.19 | 0.67 | **1.65*** | 0.76 |
| female | 3.77 | 0.34 | 2.50 | 0.70 | 2.52 | 0.41 | **1.21** | 0.26 |
| Perceived health status |  |  |  |  |  |  |  |  |
| poor to reasonable health | 3.94 | **0.06*** | 2.00 | 0.47 | 2.65 | 0.58 | 1.41 | 0.36 |
| good to excellent health | 3.72 | **0.42** | 2.57 | 0.75 | 2.36 | 0.48 | 1.32 | 0.47 |
| Level of education |  |  |  |  |  |  |  |  |
| lower educational level | 3.73 | **0.55*** | 2.48 | 0.70 | 2.43 | 0.54 | 1.33 | 0.38 |
| higher educational level | 3.80 | **0.20** | 2.45 | 0.78 | 2.41 | 0.49 | 1.35 | 0.51 |
| Healthcare stage |  |  |  |  |  |  |  |  |
| on waiting list | 3.80 | 0.16 | 2.40 | 1.04 | 2.40 | 0.44 | 1.22 | **0.17*** |
| undergone surgery/again on waiting list | 3.76 | 0.39 | 2.47 | 0.70 | 2.42 | 0.52 | 1.35 | **0.48** |

M: range 1- 4

For differences in M between subgroups, we tested if the interaction term (based on the indicator-variable and subgroup-variable) had a significant (P<0.01) regression coefficient. Differences in VAR between subgroups were tested with Wald Statistics.

* P<0.01

## Table S3 - Mean scores and variances of the clinical performance indicators for the different patient subgroups

| **Patient subgroup** | **Procedures preventing thrombosis** | | **Information provision before surgery** | | **Occurrence/prevention deep wound infections** | | **Registration of complications** | | **Transfusion of homologous blood** | | **Registration of orthopaedic implants** | |
| --- | --- | --- | --- | --- | --- | --- | --- | --- | --- | --- | --- | --- |
|  | M | VAR | M | VAR | M | VAR | M | VAR | M | VAR | M | VAR |
| Search and Selection behaviour |  |  |  |  |  |  |  |  |  |  |  |  |
| less extensive | 4.52 | 1.47 | 4.46 | 2.32 | 4.04 | 2.15 | 3.26 | 2.34 | 2.81 | 2.04 | 1.91 | 1.86 |
| more extensive | 4.18 | 1.94 | 4.19 | 3.23 | 4.26 | 1.93 | 3.03 | 1.82 | 3.22 | 2.55 | 2.05 | 2.10 |
| Age |  |  |  |  |  |  |  |  |  |  |  |  |
| up to 65 years | 4.24 | 1.90 | 4.38 | 3.16 | 4.34 | 1.94 | 3.08 | 2.03 | 2.63 | 1.78 | 2.26 | 2.11 |
| 65 years or older | 4.55 | 1.39 | 4.32 | 2.17 | 3.88 | 2.10 | 3.26 | 2.24 | 3.38 | 2.57 | 1.62 | 1.57 |
| Gender |  |  |  |  |  |  |  |  |  |  |  |  |
| male | 4.32 | 1.65 | 4.71 | 1.78 | 4.00 | 2.07 | 3.25 | 1.83 | 2.74 | 2.19 | 1.86 | 2.12 |
| female | 4.41 | 1.71 | 4.19 | 3.04 | 4.19 | 2.06 | 3.13 | 2.27 | 3.08 | 2.29 | 2.02 | 1.89 |
| Perceived health status |  |  |  |  |  |  |  |  |  |  |  |  |
| poor to reasonable health | 4.67 | 1.56 | 4.67 | 2.33 | 3.61 | 1.79 | 2.83 | 2.25 | 3.44 | 2.02 | 1.78 | 1.40 |
| good to excellent health | 4.31 | 1.70 | 4.27 | 2.77 | 4.26 | 2.06 | 3.24 | 2.08 | 2.86 | 2.28 | 2.01 | 2.09 |
| Level of education |  |  |  |  |  |  |  |  |  |  |  |  |
| lower educational level | 4.40 | 1.38 | 4.56 | 2.25 | 4.02 | 2.12 | 3.14 | 1.84 | 2.98 | 2.61 | 1.83 | 1.90 |
| higher educational level | 4.36 | 1.95 | 4.18 | 3.03 | 4.22 | 2.01 | 3.18 | 2.39 | 2.98 | 2.02 | 2.08 | 1.99 |
| Healthcare stage |  |  |  |  |  |  |  |  |  |  |  |  |
| on waiting list | 4.80 | 1.16 | 4.70 | 2.01 | 3.90 | 2.09 | 3.10 | 2.09 | 2.90 | 1.69 | 1.60 | 1.04 |
| undergone surgery/again on  waiting list | 4.33 | 1.73 | 4.31 | 2.78 | 4.16 | 2.06 | 3.17 | 2.14 | 2.99 | 2.36 | 2.01 | 2.06 |

M: range 1-6

For differences in M between subgroups, we tested if the interaction term (based on the indicator-variable and subgroup-variable) had a significant (P<0.01) regression coefficient. Differences in VAR between subgroups were tested with Wald Statistics.

## Table S4 - Mean scores and variances of the indicators about hospital services for the different patient subgroups

| **Patient subgroup** | **Specialist areas of orthopaedist** | | **Information provision approach** | | **Contact hospital after hospital discharge** | | **Number of surgeries adults** | | **Number of orthopaedists** | | **Group-hospital admission** | | **Number of surgeries children** | |
| --- | --- | --- | --- | --- | --- | --- | --- | --- | --- | --- | --- | --- | --- | --- |
|  | M | VAR | M | VAR | M | VAR | M | VAR | M | VAR | M | VAR | M | VAR |
| Search and Selection behaviour |  |  |  |  |  |  |  |  |  |  |  |  |  |  |
| less extensive | 5.30 | 3.39 | 5.00 | 2.70 | 4.83 | 1.51 | 3.98 | 3.75 | 3.61 | 3.27 | 3.54 | 3.47 | 1.70 | 1.00 |
| more extensive | 5.68 | 2.64 | 4.45 | 2.46 | 4.08 | 2.65 | 4.97 | 3.92 | 4.16 | 2.50 | 2.68 | 2.27 | 1.95 | 1.68 |
| Age |  |  |  |  |  |  |  |  |  |  |  |  |  |  |
| up to 65 years | 5.66 | 3.14 | 4.82 | 2.47 | 4.52 | 2.33 | 4.36 | 3.87 | 3.60 | 2.60 | 3.02 | 2.98 | 2.02 | **1.74*** |
| 65 years or older | 5.21 | 2.98 | 4.71 | 2.92 | 4.52 | 1.87 | 4.44 | 4.30 | 4.12 | 3.39 | 3.39 | 3.31 | 1.54 | **0.64** |
| Gender |  |  |  |  |  |  |  |  |  |  |  |  |  |  |
| male | 5.18 | 2.58 | 5.07 | 2.92 | 4.43 | 2.46 | 4.54 | 3.75 | 3.54 | 4.11 | 3.43 | 2.82 | 1.82 | 1.08 |
| female | 5.58 | 3.31 | 4.64 | 2.51 | 4.56 | 1.96 | 4.33 | 4.19 | 3.97 | 2.50 | 3.08 | 3.28 | 1.79 | 1.40 |
| Perceived health status |  |  |  |  |  |  |  |  |  |  |  |  |  |  |
| poor to reasonable health | 5.56 | 3.47 | 4.72 | 2.53 | 4.33 | 2.89 | 4.88 | 2.81 | 4.33 | 2.44 | 2.50 | 1.69 | 1.59 | **0.60*** |
| good to excellent health | 5.43 | 3.03 | 4.78 | 2.71 | 4.57 | 1.92 | 4.28 | 4.28 | 3.72 | 3.10 | 3.36 | 3.38 | 1.85 | **1.45** |
| Level of education |  |  |  |  |  |  |  |  |  |  |  |  |  |  |
| lower educational level | 5.43 | 3.01 | 4.81 | 3.11 | 4.86 | 1.88 | **3.59*** | 3.66 | 3.83 | 3.14 | 3.63 | 3.74 | 1.78 | 1.05 |
| higher educational level | 5.48 | 3.21 | 4.74 | 2.31 | 4.24 | 2.14 | **5.06** | 3.42 | 3.84 | 2.93 | 2.82 | 2.39 | 1.82 | 1.51 |
| Healthcare stage |  |  |  |  |  |  |  |  |  |  |  |  |  |  |
| on waiting list | 5.40 | 1.44 | 5.40 | 1.64 | 4.70 | 1.61 | 4.30 | 4.61 | 3.70 | 3.81 | 2.90 | 2.89 | 1.60 | **0.44*** |
| undergone surgery/again on waiting  list | 5.46 | 3.32 | 4.70 | 2.75 | 4.50 | 2.18 | 4.41 | 3.99 | 3.85 | 2.93 | 3.22 | 3.19 | 1.83 | **1.40** |

M: range 1-7

For differences in M between subgroups, we tested if the interaction term (based on the indicator-variable and subgroup-variable) had a significant (P<0.01) regression coefficient. Differences in VAR between subgroups were tested with Wald Statistics.

* P<0.01
